# The Effect of Advanced Platelet-Rich Fibrin Plus (A-PRF+) on Graft Stability in Dental Implants and Alveolar Ridge Augmentation Procedures: A New Low-Speed Standardized Centrifugation Protocol

**DOI:** 10.3390/dj12110349

**Published:** 2024-10-31

**Authors:** Benjamin Walch, Andreas Kolk, Dominik Scheibl, Maria Guarda, Sarah Christine Maier, Lena Denk

**Affiliations:** 1 Department of Oral and Maxillofacial Surgery, Medical University of Innsbruck, A-6020 Innsbruck, Austria; 2 Institute of Clinical Epidemiology, Public Health, Health Economics, Medical Statistics and Informatics, Medical University of Innsbruck, A-6020 Innsbruck, Austria

**Keywords:** platelet-rich fibrin, alveolar bone loss, alveolar ridge augmentation, alveolar bone grafting, bone substitutes

## Abstract

**Background**: Platelet-rich fibrin (PRF) is a concentrate derived from autologous blood, containing platelets, fibrin, and growth factors (GF) obtained through centrifugation. PRF can be mixed with bone replacement material to form sticky bone, which is then introduced into the desired area for stabilizing and graft-covering. Depending on the centrifugation protocol, the effectiveness of the end products can vary. This controlled clinical study examines the impact of our established PRF protocol in alveolar augmentation and dental implant placement on vertical bone loss. **Materials and Methods**: A total of 362 implants were performed in 170 patients at the Department of Oral and Maxillofacial Surgery at the University Hospital Innsbruck between 2018 and 2021. After accounting for lost implants, we retrospectively evaluated a radiological vertical bone loss exceeding 1 mm in the first year as the primary endpoint. **Results**: The use of PRF was significantly associated with vertical bone loss > 1 mm (OR = 0.32, 95% CI (0.13–0.81), *p* = 0.016). There were no significant associations between PRF and the number of implants lost, the type of augmentation, or bone loss. **Discussion**: A-PRF+ sticky bone grafts, when combined with bone graft materials, show reduced resorption rates, indicating their potential to enhance graft stability in oral implantology. Our data indicate that the Medifuge MF 100 (Silfradent srl, Forlì, Italy) is effective in producing A-PRF+.

## 1. Introduction

Platelet-rich fibrin (PRF) is an autogenous concentrate obtained by centrifugation of blood. It represents a further development of platelet-rich plasma (PRP), and differs from it in the increased fibrin concentration, which is about ten times the physiological concentration. PRF is produced without the addition of anticoagulant adjuvants and is centrifuged at high forces. One crucial benefit of PRF is the formation of a fibrin matrix, which gradually releases growth factors [1,2,3,4]. Leukocyte content and fibrin architecture are two key essential qualities of platelet concentrates.

Following the development of rapid-spun L-PRF by Choukroun in the early 2000s, advanced PRF (A-PRF) was developed to increase the cell content in the blood concentrate. This was achieved by decreasing the centrifugation speed to 1300 RPM and thus the relative centrifugation force to 200× *g*. Later, advanced PRF plus (A-PRF+) was developed by also decreasing the centrifugation duration [5,6]. Besides those, a plethora of PRF variants can be found in the literature. Horizontal platelet-rich fibrin (H-PRF) is produced by a more effective horizontal centrifugation method. I-PRF is created using very low relative centrifugal forces by spinning at 700 RPM for 3 min producing a liquid injectable PRF without the use of anticoagulants [6].

Not only the way in which the PRF is spun, but also the methods used for harvesting and the types of tubes used can affect the final product. Concentrated PRF (C-PRF), although produced using protocols similar to those of the original L-PRF, is distinguished by a specific harvesting technique that involves collecting the buffy coat layer directly above the red blood cells. Likewise, Titanium-PRF (T-PRF) employs centrifugation values similar to those used for the original L-PRF but is specifically produced in titanium tubes. This unique process is intended to enhance platelet activation and result in a highly organized leukocyte matrix [7,8].

Of these new PRF variants, A-PRF+ might be one of the most promising blood products. The structural matrix of A-PRF+ allows for a slow, sustained release of growth factors, resulting in prolonged biological activity. Additionally, the lower centrifugation g-forces help preserve more cells, leading to a higher concentration of growth factors resulting in prolonged biological activity. Additionally, the lower centrifugation g-forces help preserve more cells, leading to a higher concentration of growth factors in general [9,10].

However, it’s important to note that even if two different devices are used to create the same PRF archetype, the resulting products may not be identical. Differences in the rotor’s angle or radius variations can affect the relative centrifugal force produced. Furthermore, some devices have built-in vibrations that can lead to resonance phenomena, potentially impacting the quality of the blood products. For these reasons, Miron et al. suggest that studies should list the following six parameters: (1) dimensions of the rotor; (2) rotor angulation for the tube holder; (3) revolutions per minute (RPM) and time; (4) RCF value calculated at either the RCF-min, RCF-clot, or RCF-max; (5) composition and size of tubes used to produce PRF; and (6) centrifugation model used [11].

Despite their differing properties, PRF products primarily function by stimulating biological wound healing through the local release of growth factors. Platelets contain a variety of cytokines, which are key inflammation and healing mediators [12].

But PRF not only aids alveolar ridge augmentation by releasing growth factors; it also improves the handling of bone grafting materials. The PRF extracted from the centrifugation tube can be mixed with bone replacement material to create sticky bone or it can be pressed to form a membrane. When used as sticky bone, it acts as a biological connector between bony particles. As a membrane, it protects and stabilizes bone grafts in ridge augmentation by providing additional cover during the healing process [13,14].

The data suggests that A-PRF+ in particular has a positive effect on alveolar ridge augmentation and dental implantation. However, evidence indicates that the efficacy of PRF can vary based on the centrifugation protocol and mode of application [15]. This study aims to explore the promising but limited data in the literature regarding the use of A-PRF+.

Specific objectives:

− To evaluate the impact of A-PRF+ on marginal bone loss after alveolar ridge augmentation and dental implant placement.
− To provide detailed information about the equipment used and the preparation parameters, as requested in recent publications.
− Define future perspectives for the research on A-PRF+.

## 2. Materials & Methods

This study was approved on 5 August 2021 by the Ethics Committee of the Medical University of Innsbruck (Approval No. 1133/5 August 2021) and was conducted in accordance with the principles of the Declaration of Helsinki. The STROBE guidelines were adhered to in the preparation and writing of this work.

439 patients who had dental implant surgery performed at the Department of Oral and Maxillofacial Surgery at the University Hospital Innsbruck between 2018 and 2021 were evaluated for possible inclusion in this study. In those patients, a total of 921 implants were placed. On average, patients received 2.1 dental implants, with a range between one and ten. Out of the 921 implants, it was possible to determine whether augmentation occurred in 879 cases. Comparable types of alveolar ridge augmentations using autogenous bone or particulate xenogenic bone were performed in 429 of these cases. Information on the use of PRF was available for 362 implants in 170 patients. Those patients were included in this study. Written informed consent was obtained from the patient in accordance with standard procedures.

**Figure 1:**
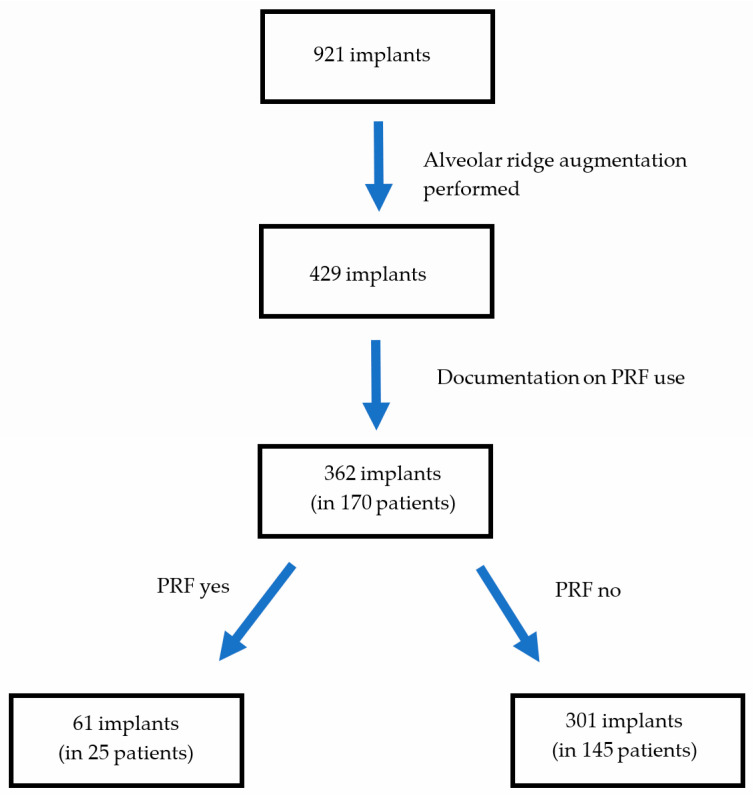
Patient collection flowchart.

The intervention group consisted of 61 implants in 25 patients, where augmentation was performed using A-PRF+. The control group comprised the remaining 301 implants in 145 patients, which were placed after bone augmentation without the use of A-PRF+ (Figure 1).

### 2.1. Risk Factors

In addition to the use of PRF, further data were collected from the hospital information system KIS Powerchart Millenium (Oracle Cerner, Kansas City, MI, USA) documentation: person-specific information such as date of birth, gender, smoking status, periodontitis, and systemic diseases like diabetes and osteoporosis were documented. There were no patients with a history of medication-related osteonecrosis of the jaw or radiotherapy in the head and neck area. Other risk factors were excluded due to a lack of documentation or because they were not applicable to our patient cohort. Selection of risk factors as covariates for the GEE model was based on clinical relevance [16,17,18].

### 2.2. Augmentation, Implant Type and Dental Restorations

**Figure 2:**
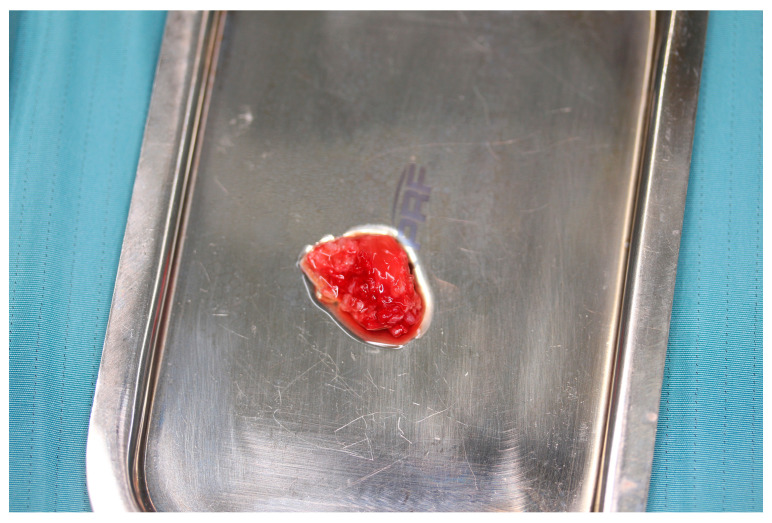
A-PRF+ sticky bone.

Additionally, implant-related values were reported. Recorded data included type, length, and diameter of the implant, implantation time, indication, and whether augmentation was performed. This included the type of augmentation, such as whether autogenous bone (Figure 2) or particulate xenogenic bone (Bio-Oss, Geistlich, Germany; Cerabone, Regedent, Germany) was used for onlay or inlay grafting, and if PRF was applied. Three types of Straumann Bone Level Implants were used: the Straumann Bone Level Implant, Straumann Bone Level Tapered BLT, and Straumann BLX. Additionally, two Tissue Level Implants were used: the Straumann Tapered Effect Implant and Straumann Standard Plus (Straumanngroup, Basel, Switzerland). Prosthetic restorations were done by an external dentist, a clinical dentist at the Department of Oral and Maxillofacial Surgery, or at the Department of Dentistry of the University Clinic of Innsbruck.

### 2.3. PRF Centrifugation Protocol

- In this study, we utilized a Medifuge MF 100 (Silfradent srl, Forlì, Italy) featuring a clotrra
- Radius of 55.55 mm and an “end of tube” radius of 83.40 mm, with a rotor angulation of 33°. For the centrifugation process, we employed glass tubes: the PV 200R for solid clots and the PV 200P with ACDA and separator gel for the liquid phase. Following the A-PRF+ protocol, we centrifuged at 1680 rpm for 8 min, achieving a relative centrifugal force (RCF) of 177 *g* (Table 1).

### 2.4. Bone Loss and Radiological Bone Measurements

The data was collected through measurements of CBCT scans, panoramic X-rays, and dental X-rays taken at the time of implantation and comparing the equivalent radiological images at the routine yearly checkups.

**Figure 3:**
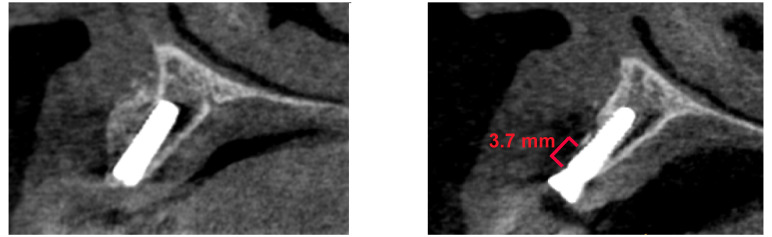
3.7 mm vertical bone loss of buccal augmentation.

The radiological measurement was performed at the point of the most significant resorption. After accounting for lost implants, we retrospectively evaluated a radiological vertical bone loss exceeding 1 mm in the first year as the primary endpoint (Figure 3).

### 2.5. Statistical Analysis

The data are listed in Microsoft Excel (Microsoft, Version 2406, Redmond, WA, USA), and the Statistical Package for the Social Sciences (SPSS Version 21.0; SPSS, IBM, Chicago, IL, USA) is used for descriptive statistics. Data were presented using descriptive methods. Absolute and relative frequencies for qualitative variables and mean ± standard deviation for quantitative variables were calculated. A binary logistic regression was performed using generalized estimating equations (GEE) to account for dependencies of implants within patients (362 implants, 170 patients). First, a univariable model was conducted and secondly, the model was adjusted for possible confounders (age, sex, osteoporosis, periodontitis, and smoking). To account for missing values (smoking, osteoporosis, periodontitis) multiple imputations were performed. Further analyses of possible associations were also performed using univariable binary logistic regression models using GEE.

## 3. Results

### 3.1. Bone Loss

**Figure 4:**
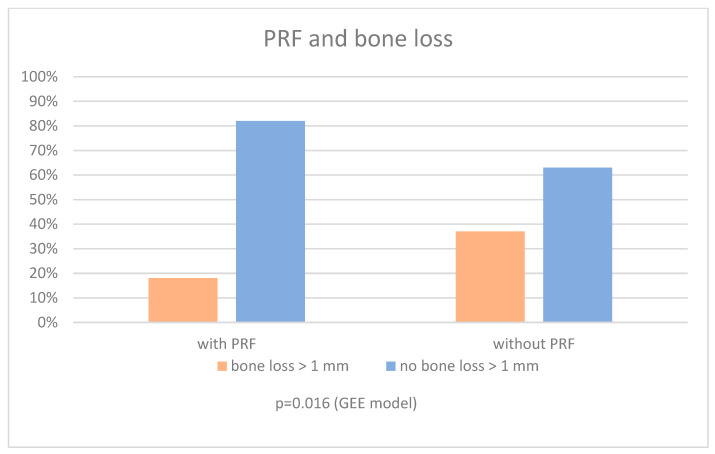
Bar chart of PRF and Bone loss, *n* = 277 implants.

In the univariable model, PRF was significantly associated with decreased vertical bone loss > 1 mm (OR = 0.37, 95% CI (0.17–0.82), *p* = 0.014). Even after adjusting for possible confounders (age, sex, osteoporosis, periodontitis, and smoking), PRF continued to show a significant association with bone loss > 1 mm (OR = 0.32, 95% CI (0.13–0.81), *p* = 0.016 (Figure 4 and Table 2).

### 3.2. Implant Loss and Augmentation Method

PRF did not show a significant effect on implant loss, which occurred in 23/295 (7.8%) cases (OR = 1.26, 95% CI (0.29–5.56), *p* = 0.756). The type of augmentation used (37.8% autologous vs. 62.2% xenogenic) showed no significant association (OR = 1.14 (0.64–2.04), *p* = 0.650) with bone loss. Univariable logistic regression analyses (GEE) showed no significant association between bone loss and the following parameters: type, length, diameter of the implant, implantation time, and indication.

### 3.3. Risk Factors

The risk factors age and sex were distributed relatively evenly across the two groups. Smoking (34.0% vs. 28.8%), periodontitis (85.7% vs. 56.3%), diabetes (6.7% vs. 1.7%), and osteoporosis (13.3% vs. 1.3%) occurred more frequently in the PRF group (Table 3).

### 3.4. Post Hoc Power Analysis

We performed a post-hoc power analysis using the sample sizes and the proportions of bone loss > 1 mm observed in our study (without PRF: 37%, 84/227 vs. with PRF: 18%, 9/50). This analysis revealed a statistical power of 76% to detect the difference in bone loss as statistically significant on the 5% significance level.

## 4. Discussion

PRF contains a variety of growth factors and serves as a mechanical biomaterial, stabilizing bone grafts during wound healing [14]. Due to its properties, PRF can also be considered a potent, locally applied autogenous combination drug. Some of the growth factors released by the thrombocytes contained in PRF are available individually as recombinant FDA-approved drugs, demonstrating positive effects when utilized on their own for alveolar ridge preservation and bone augmentation [19,20,21].

While the roles of the cell types involved and the molecular pathways of the GFs are well understood, their exact clinical benefit in alveolar ridge augmentation is not fully researched. In our controlled clinical study, we highlight the significant benefits of combining PRF with bone augmentation in a standardized A-PRF+ protocol. Even after adjusting for possible confounders (age, sex, osteoporosis, periodontitis, and smoking) we continued to observe a significant association with decreased vertical bone loss. Our results align with the recent literature, which also validates A-PRF+‘s benefits in hard tissue regeneration, while there is already substantial evidence supporting the biological effectiveness of PRF in soft tissue management [15,22].

However, results concerning hard tissue vary among studies. Discrepancies in the data may be due to variations in the PRF preparation itself. The authors suggest examining the specific protocols used for PRF preparation. Most studies describe the used PRF in terms of known standards of platelet-rich fibrin (PRF) like L-PRF, A-PRF, A + PRF, or I-PRF. Nonetheless, the precise composition of growth factors in the same PRF preparation can vary significantly based on the centrifugation protocol used and may even differ depending on the design of the centrifugation devices themselves [15].

Ehrenfest et al. examined L-PRF obtained from four different centrifuges with standardized parameters: g-force and centrifugation time. Consequently, the only remaining variable among the four products was the centrifuge machine itself. Upon completion of blood centrifugation across these different machines, variations were observed in the L-PRF clots regarding their weight, volume, fibrin architecture, and cellular content. The lower cell content observed in some of the L-PRFs is attributed to the intrinsic vibration of the machines, resulting in resonance phenomena. According to the authors those phenomena lead to blood products with compromised and nearly destroyed cell populations when the standard L-PRF protocol is employed. This study suggests that not only do centrifugation devices yield different products, but some may also be unsuitable for producing L-PRF altogether [23].

Unfortunately, in the past, crucial details of PRF preparation were often omitted in published studies. Therefore, recent reviews have called for the precise documentation of the PRF protocols and the equipment used [15,22,24].

Miron et al. suggest studies to include the following six parameters: (1) dimensions of the rotor; (2) rotor angulation for the tube holder; (3) revolutions per minute (RPM) and time; (4) RCF value calculated at either the RCF-min, RCF-clot, or RCF-max; (5) composition and size of tubes used to produce PRF; and (6) the centrifugation model used [11].

This study was performed using an A-PRF+ protocol the exact features of the centrifugation device are listed in the Section 2 (Table 1).

In our 362 implant cases, we observed a significantly lower bone loss within the first year in the A-PRF+ group, suggesting that A-PRF+ enhances the bone healing of the graft. When trying to compare our results to the literature we found none with exact matching methods of preparation; however, some authors used comparable techniques (Table 4).

Yewale et al. observed a significant decrease in vertical bone loss of the graft during healing when PRF was used in alveolar ridge augmentation. They evaluated the effect of PRF in a standard protocol for ridge augmentation and GBR using a biphasic synthetic HA and Beta-TCP particulated graft, both alone and mixed with A-PRF+, in two groups of 10 patients each. A-PRF+ was produced using a protocol of 1300 RPM for 8 min, stating 208 *g* as the resulting force. Blood samples were collected in 10 mL tubes without anticoagulants. No further details about the hardware were provided [25]. The A-PRF used along with augmentation method despite using another graft material is very similar to our standard protocol.

We believe that the observed reduction in bone loss is primarily due to the enhanced stability of the graft material, as well as the improved quality of the newly formed bone associated with the use of PRF in general and A-PRF+ in particular. This theory is strongly supported by abundant evidence from histological and mechanical studies evaluating the grafted bone after healing.

Işık et al. reported positive effects on the stability of xenogenic grafts in horizontal augmentations when enriched with platelet-rich fibrin (PRF) compared to the same bovine-derived grafts without PRF. The intervention group consisted of 20 patients with 50 implants. In those patients, the augmentation was performed using PRF-enriched bovine-derived xenografts. The control group, which included 20 patients with 48 implants, received the same type of grafts without PRF. The intervention group showed significantly less bone loss in the PRF-enriched grafts compared to the control. In this study, a modified version of injectable PRF (I-PRF) was used, created with a low centrifugal force compared to the original I-PRF (standard: centrifuged at 700 rpm (60 G) for 3 min). The exact centrifugation protocol was detailed in the study. The blood product was prepared at 700 rpm for 3 min (RCF-max~44 *g*) in 10-mL, non-coated, plastic tubes, and the angulation of the fixed-angled centrifuge device was 33° rotor angulation with a 50-mm radius at the clot and 80 mm at the maximum. The device used was the EBA20-centrifuge (Hettich, Tuttlingen, Germany) [30]. Despite not using the same device and a different centrifugation protocol, the clinical application, as well as their findings, were very similar to ours for alveolar ridge augmentation.

De Almeida et al. observed improved histological and resonance frequency outcomes during implant placement after augmentation with deproteinized bone graft alone compared to the same graft in combination with L-PRF [31]. Troedhahn reported a two-fold increase in insertion torque value in the A-PRF group compared to the control [32]. Others observed improved histological new bone formation without improved mechanical outcomes in a similar controlled trial [33]. Pichotano et al. listed their PRF centrifugation protocol as using 3000 rpm for 10 min in a Kasvi K14-0815 device (Kasvi, Curitiba, PR, Brazil). No further information on radius of the centrifuge or the g-force was given and thus the latter cannot be calculated. This observation was replicated in a systematic review that included five controlled clinical trials using various augmentation materials, including allogeneic, xenogenic, and synthetic types. The review referred to the L-PRF products being fabricated using Choukroun’s procedure, with centrifugation times ranging from 10 to 12 min, rpm values between 2700 and 3000, and mean centrifugal forces of 300–400 *g*. Two devices were specified: a table centrifuge (Nüve Laboratory Equipment, NF200, Ankara, Turkey) and an IntraSpin centrifuge (IntraSpin, USA) [34]. In conclusion, the data reporting positive effects of PRF in general are plentiful. However the protocols reported are mostly incomplete [31,33,34] and sometimes not listed at all [32].

Clinical use data on A-PRF+ compared to A-PRF on the contrary is limited, as it is a recent invention and among the newer blood products. Clark observed decreased bone loss in the A-PRF+ and A-PRF groups compared to the blood clot; however, no difference in effectiveness between the concentrates was noted. They used a standard method to prepare the A-PRF, but there were no details provided about the centrifugation device or the A-PRF+ preparation [28]. In vitro experiments indicate that A-PRF+ centrifugation protocols, by reducing g-force, lead to better cell preservation, greater release of growth factors, and may induce more cell migration during the healing period [9,10,26,27]. Kosmidis found A-PRF+ treated osteocytblast-like cell lines to induce more mineralization and increased calcium production [29]. Even though studies have shown promising results, the role of A-PRF+ in alveolar ridge augmentation remains unclear. The findings of our investigation suggest that our protocol is highly effective.

However, the limitations of our study must be acknowledged. Foremost, our research is limited by a lack of comparative studies specifically on A-PRF+ and its role in alveolar ridge augmentation in the current literature, since A-PRF+ is one of the newer PRF variants. We tried to compensate for this by laying out a rationale from the existing data to other comparable PRF variants and augmentation methods. Also, in vitro studies on the positive properties of A-PRF+ could hint to a reason for our observed positive clinical efficiency in preserving bone grafts. There were no reports on the Medifuge MF 100 (Silfradent srl, Forlì, Italy) and A-PRF+. Thus, evaluating its capacity to create high-quality A-PRF+ from the literature was not possible.

There is promising data on the healing capacity of A-PRF+ in in vitro studies and other applications [35,36,37]; however, further research is needed to gather sufficient evidence regarding the effectiveness of A-PRF+ in alveolar ridge augmentation. We want to emphasize that, despite our best efforts to collect the reported data accurately, retrospective studies always carry the risk of bias.

We observed a discrepancy in participant numbers between the PRF and non-PRF groups, which may have been caused by a limited supply; only one centrifuge was available for multiple operating rooms. This situation may also explain why conditions such as osteoporosis and periodontitis were more prevalent in the PRF group, as clinicians might have prioritized the use of additional PRF when such diagnoses were documented in the patient’s history. Moreover, there was quite a bit of variability in the data, including differences in the augmentation materials used. Nevertheless, both autogenous and deproteinized xenogenic bone grafts were evenly distributed between the intervention and control groups, and we observed no statistically significant difference in vertical bone loss among the different graft materials.

By measuring all documented patients and implant sites, this report provides real-world data from our clinical practice detailing numerous cases of A-PRF+ use in alveolar ridge augmentation and its effects on these procedures.

## 5. Conclusions

When combined with particulate autologous or xenogenic bone graft materials, A-PRF+ sticky bone grafts demonstrated reduced resorption rates, suggesting that A-PRF+ may be a viable option for enhancing graft stability in oral implantology. Our data indicate that the Medifuge MF 100 (Silfradent srl, Forlì, Italy) effectively produces A-PRF+. However, additional research is needed to evaluate this device and the new low-speed centrifugation method within standardized protocols. Future trials should systematically compare this low-speed centrifugation technique, including its associated hardware and parameters, against established methods.

## Figures and Tables

**Table 1:** PRF Centrifugation protocol.

Centrifuge	type	MEDIFUGE MF 100 (Silfradent srl, Forli, Italy)
	radius	55.55 mm (clot level) 83.40 mm (end of tube)
	rotor angulation	33°
Tube	solid clot	PV 200R 10 mL glass tube
	liquid phase	PV 200P 8 mL glass tube ACDA + separator gel
A-PRF+ protocol	time	8 min
	speed	1680 rpm

**Table 2:** Binary logistic regression using GEE.

	Univariable Model, *n* = 277	Multivariably Adjusted Model, *n* = 277
	OR (95% CI), *p*-Value	OR (95% CI), *p*-Value
PRF	0.37 (0.17–0.82), 0.014	0.32 (0.13–0.81), 0.016
Age		1.01 (0.99–1.03), 0.508
Sex		1.03 (0.56–1.89), 0.931
Smoking		2.14 (0.93–4.91), 0.071
Periodontitis		0.99 (0.52–1.91), 0.980
Osteoporosis		1.15 (0.20–6.58), 0.876

**Table 3:** Risk factor distribution.

	All Implants *n* = 362	With PRF *n* = 61	Without PRF *n* = 301
Patients	170	25	145
Age	53.2 (16.3)	56.0 (17.7)	52.7 (16.0)
Female, *n* (%)	200 (55.2)	34 (55.7)	166 (55.1)
Smokers, *n* (%)	78 (29.8), *n* = 262	16 (34.0), *n* = 47	62 (28.8), *n* = 215
Periodontitis, *n* (%)	33 (60.0), *n* = 55	6 (85.7), *n* = 7	27 (56.3), *n* = 48
Diabetes, *n* (%)	9 (2.5), *n* = 360	4 (6.7), *n* = 60	5 (1.7), *n* = 300
Osteoporosis, *n* (%)	12 (3.3), *n* = 361	8 (13.3), *n* = 60	4 (1.3), *n* = 301

**Table 4:** Literature on A-PRF+.

	Study Design	PRF Preparation (Speed, Time, RCF)	Device	In Accord with Our Findings
Pitzurra et al. [9]	In-vitro study	n.a. 8 min 208 *g*	Duo centrifuge (Process for PRF™, Nice, France)	Yes
Yewale et al. [25]	Clinical study	1300 rpm 8 min 208 *g*	n.a.	Yes
Simoes-Pedro et al. [26]	In-vitro study	1500 rpm 8 min 126 *g*	IntraSpin™ centrifugation device (Intra-Lock, Boca Raton, FL, USA)	Yes
El Bagdadi et al. [10]	In-vitro study	1300 rpm 8 min 208 *g*	Duo centrifuge (Process for PRF™, Nice, France)	Yes
Fujioka-kobayashi et al. [27]	In-vitro study	1300 rpm 8 min 200 *g*	Duo centrifuge (Process for PRF™, Nice, France)	Yes
Clark et al. [28]	Clinical study	1300 rpm 8 min 200 *g*	n.a.	Yes
Kosmidis et al. [29]	In-vitro study	n.a. 8 min 208 *g*	Duo centrifuge (Process for PRF™, Nice, France)	Yes

## Data Availability

The data presented in this study are available on request from the corresponding author due to privacy, legal and ethical reasons.
